# Culture-Dependent and Molecular Characterization of Bacteria Associated with Banana Fruits in Puerto Rico 

**DOI:** 10.17912/micropub.biology.002342

**Published:** 2026-09-25

**Authors:** Alexis Manfredy Dedós, Alanis G. Carmona Beltrán, Lilli R. Hanson, Andrea M. Núñez Ortiz, Jomitsy N. Santana Rivera, Naiomy Ríos Colón, Jesus M. Ramos Arroyo, Christopher A. Sambolín-Pérez

**Affiliations:** 1 BIOL3105.General Microbiology, Department of Science and Technology, Inter American University of Puerto Rico, Barranquitas, Puerto Rico, USA; 2 Department of Natural Sciences and Mathematics, Inter American University of Puerto Rico, Bayamón, Puerto Rico, USA; 3 Department of Biological Sciences, California State University Stanislaus, Turlock, California, USA; 4 Department of Science and Technology, Inter American University of Puerto Rico, Barranquitas, Puerto Rico, USA; 5 MICR4010.Microbial Ecology, Department of Science and Technology, Inter American University of Puerto Rico, Barranquitas, Puerto Rico, USA; 6 Institute of Sustainable Biotechnology, Inter American University of Puerto Rico, Barranquitas, Puerto Rico, USA

## Abstract

The genus *Musa* spp. encompasses significant global food crops, including bananas and plantains, which play an essential role in the economies, nutrition, and culinary practices of developing countries. Nonetheless, *Musa* spp. face threats from abiotic and biotic stressors, including phytopathogens. Consequently, exploring cultivable bacteria on fruits is vital for understanding bacterial ecological dynamics. Isolates were characterized using culture-dependent techniques: 16S rDNA barcoding and functional prediction, phylogenetic analysis, and morphological approaches. This exploratory study identified three isolates associated with plants and the environment. Future research should conduct larger surveys and genomic analyses around the island to understand bacterial ecology in bananas.

**Figure 1. Phenotypic and Molecular Characterization of Bacteria Isolated from Symptomatic Banana Fruits:** Overview of the schematic representation of the study workflow and symptomatic banana fruits obtained from a local market in Barranquitas, Puerto Rico (A). Purified bacterial isolates, Gram-staining morphology of the three isolates: (1) *Klebsiella variicola* , Gram-negative; (2) *Pantoea dispersa* , Gram-negative; and (3) *Bacillus altitudinis* , Gram-positive (B); all tested positive for catalase. Maximum likelihood phylogenetic analysis was conducted based on the 16S rRNA gene of bacterial isolates, along with known references for each genus. A bootstrap consensus tree was inferred from 1,000 replicates. The values shown next to the corresponding nodes represent the percentage of replicate trees in which the associated taxa clustered together. Isolates obtained in this study are designated “BN,” and the tree was rooted using *Micrococcus luteus* as the outgroup (C). Bubble plot of predicted ecological roles and metabolic potential of isolates using FAPROTAX; predictions were inferred from the evolutionarily nearest taxonomic groups. Ecological roles associated with humans and plants are highlighted in the green and blue boxes, respectively (D). Created in BioRender. Sambolin, C. (2026). https://BioRender.com/btzovxd. **Table 1.** Basic Local Alignment Search Tool (BLAST) analysis of 16S rDNA barcoding of isolated bacteria from banana fruit.

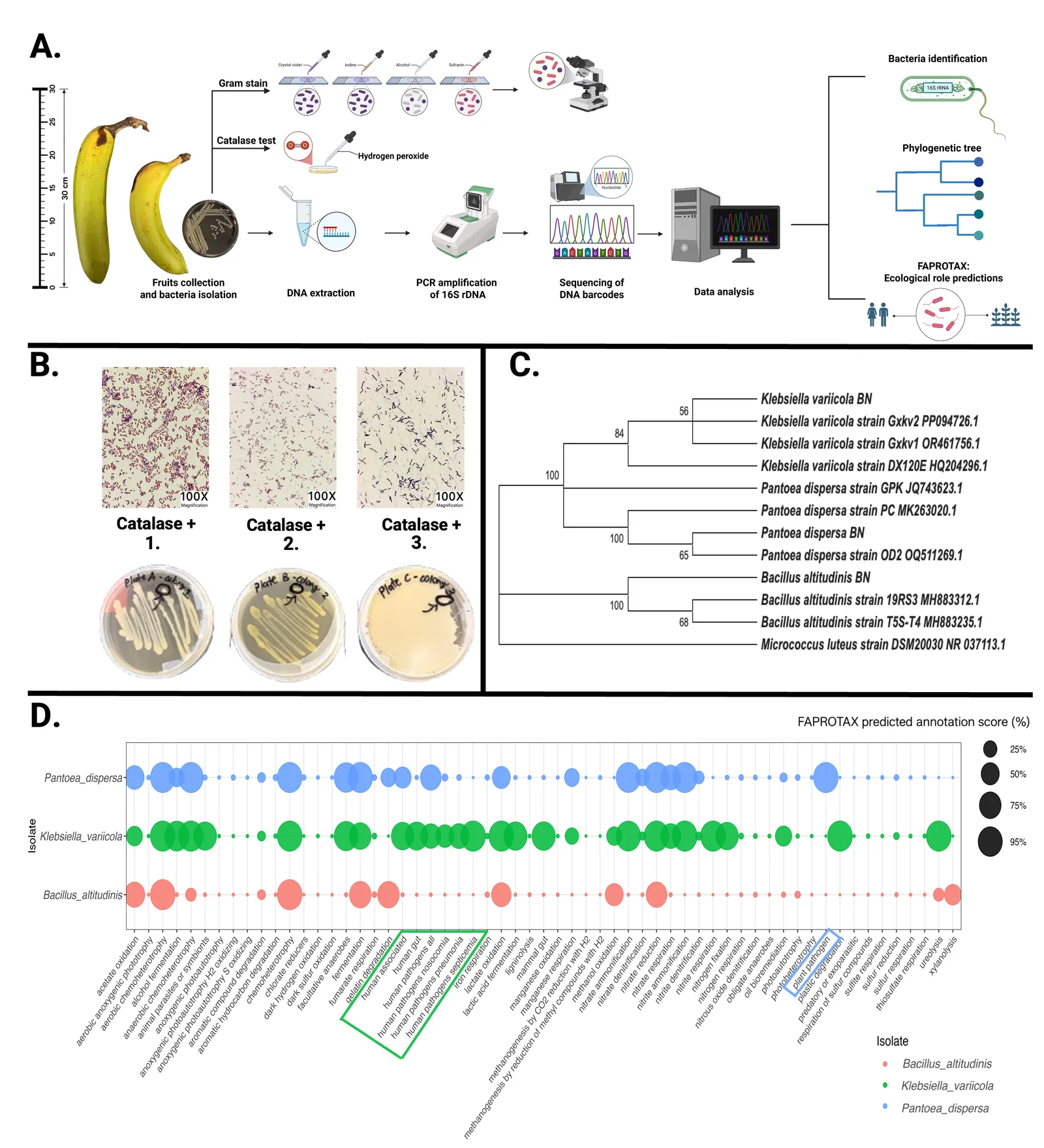



**Table:** 

Host	Scientific Name	GenBank Accessions	Query Cover %	Identity %	Reference Accession
Banana	Klebsiella variicola	PZ624115	100	100	CP153076.1
Banana	Pantoea dispersa	PZ624116	100	99.93	MT826213.1
Banana	Bacillus altitudinis	PZ624117	100	100	CP124831.1

## Description

Bananas and plantains, belonging to the genus *Musa* spp., are globally important food crops because of their nutritional value, economic impact, culinary uses, and role in sustainability for developing countries (FAO, 2022; Leonel et al., 2024). As such, Latin America and the Caribbean (LAC) account for over 60% of banana and over 70% of plantain exports on international markets (Blomme et al., 2017), along with providing food security and sustainability to the regions that consume around 20 million tons (Dita et al., 2013; Sambolín-Pérez et al., 2026). Beyond their significance, *Musa* spp. are increasingly threatened by abiotic factors that are becoming more persistent in Latin America and the Caribbean, especially in Puerto Rico (hurricanes Maria, 2017; Fiona, 2022) and in The Bahamas, Jamaica, Haiti, and the Dominican Republic (hurricane Melissa, 2025), which have experienced several hurricanes directly impacting agriculture (IICA, 2025; Rodríguez-Cruz et al., 2022; Rodríguez-Cruz & Niles, 2018). In addition to abiotic stresses, biotic factors such as phytopathogens have raised concerns about agricultural productivity across LAC. These pathogens mainly include fungi and bacteria such as *Fusarium oxysporum* f. sp. *cubense* , *Mycosphaerella fijiensis* , and *Ralstonia solanacearum* , causing diseases such as Fusarium wilt, Black Sigatoka, and Moko, respectively (Blomme et al., 2017; Dale et al., 2017; Ploetz, 2006; Sambolín et al., 2025). Consequently, there is growing interest in conducting surveys on these vital global food crops to identify, characterize, and develop preventive strategies and biological controls against such phytopathogens.

Hence, this study furthered the existing survey of banana bacterial diseases in Puerto Rico by characterizing bacteria isolated from symptomatic tissues of local banana fruits. Combining molecular techniques with conventional methods enabled the identification and phenotypic profiling of these bacteria. Therefore, this study offers valuable insights into the cultivable bacterial diversity within tissues exhibiting fruit disease symptoms, providing essential information for local agriculture and the wider LAC regions. This is particularly significant given that pathogens can easily disperse across regions via various vectors, including tools, water, infected plant materials, and insects, among others (Hayes et al., 2022; Lei et al., 2025; Roels et al., 2005).

Three bacterial strains were successfully isolated employing culture-dependent methods on banana fruit tissues showing disease symptoms ( Fig. 1A ), comprising two Gram-negative and one Gram-positive bacteria. All isolates tested positive for catalase activity and produced distinctive colonies, which ranged in color from white, yellow, to off-white ( Fig. 1B ). Molecular approaches were applied to identify the isolates, which produced near full-length 16S rDNA sequences (~1400 bp). Following analysis using NCBI BLAST, isolates were identified presumptively as *Klebsiella variicola* , *Pantoea dispersa* , and *Bacillus altitudinis* with 100% query coverage and over 99.9% identity (Table 1). Phylogenetic analysis employing Maximum Likelihood demonstrated that the bacterial isolates clustered within their respective reference classes, as indicated by bootstrap values of 100% ( Fig. 1C ). Conversely, some within-clade relationships exhibited lower support, showing bootstrap values ranging from 56% to 84%. Nonetheless, this analysis was performed to confirm the clustering of the isolates with their corresponding species references and, consequently, their respective clades. Additionally, a 16S rDNA amplicon sequence-based functional prediction tool was employed to gain insights into the potential roles of the isolates. The Functional Annotation of Prokaryotic Taxa (FAPROTAX) tool has previously demonstrated its effectiveness in predicting bacterial communities within terrestrial ecosystems (Sansupa et al., 2021). A total of 59 potential functions and roles were predicted across the three isolates ( Fig. 1D ). Among these, human-associated pathogens and plant pathogens were identified. Additionally, essential host and ecological roles were also identified, including aerobic chemeheterothrophy, among others. *Klebsiella* was primarily linked to human-associated roles, whereas *Pantoea* was associated with plant pathogenicity, and *Bacillus* was linked to common ecological functions such as nitrate reduction. However, rather than confirming these functional roles, we interpret the results as potential ecological roles, emphasizing the need for mechanistic validation.

*Klebsiella variicola* is a facultative anaerobic, non-motile, and Gram-negative bacterium with the capacity to grow in a broad range of temperatures, forming circular, convex, and mucoid colonies (Lin et al., 2015; Rodríguez-Medina et al., 2019). Although this bacteria has been associated with beneficial properties for plant hosts, including the capacity to fix nitrogen and promote plant growth (Lin et al., 2015), it has also demonstrated the potential to be an opportunistic plant pathogen and cause plant diseases (Loganathan et al., 2021), including plantain soft rot (Fulton et al., 2020), bulb rot disease and bacterial wilt disease in banana (Jiang et al., 2024; Toh et al., 2024), and banana sheath rot (Sun et al., 2023). Despite the lack of reports confirming the isolation or molecular characterization of *Klebsiella variicola* on *Musa* spp. in Puerto Rico, this underscores the need for further research on this bacterium, as it is considered an emerging phytopathogen (Sun et al., 2023; Toh et al., 2024). This bacterium has been recently documented in Haiti, causing plantain diseases (Fulton et al., 2020), posing a threat to agriculture in U.S. territories in the Caribbean. Additionally, this taxon has been previously associated with inter-kingdom capabilities to induce diseases such as bloodstream, respiratory tract, and urinary tract infections in humans, mastitis in bovines, and wetwood in trees (Ayin et al., 2015; Martínez-Romero et al., 2018; Rodríguez-Medina et al., 2019). Like *Klebsiella variicola* , *Pantoea dispersa* is a Gram-negative, non-spore-forming, rod-shaped bacterium that can be isolated from various ecological sources such as plant tissues, humans, and the environment (Asai et al., 2019). Similarly, *P. dispersa* is generally considered a phytopathogen and has been shown to infect plant tissues and induce disease in strawberries (Wang et al., 2025). While certain *Pantoea* species are suggested to form symbiotic relationships with plants, offering protection against phytopathogens (Duchateau et al., 2024), clinical cases remain uncommon (Asai et al., 2019; Hagiya & Otsuka, 2014; Schmid et al., 2003). Conversely, *Bacillus altitudinis* is a Gram-positive, rod-shaped bacterium (Vettath et al., 2017) and has been previously associated with banana plants (Dinesh et al., 2026). It has demonstrated both biocontrol capabilities and plant growth-promoting properties (Falcón-Piñeiro et al., 2026) and has also been proposed as a potential biofertilizer candidate due to its capacity to enhance plant growth, thereby supporting sustainable agricultural practices (Zhang et al., 2021).

Although the association of these taxa ( *K. variicola* , *P. dispersa* , and *B. altitudinis* ) with different kingdoms aligns with their ecological roles and functional potential, their behaviors involve complex interactions with their environment. Previous studies have shown that banana disease is associated with changes in the host's endophytic bacterial community, suggesting that interactions between *K. variicola* and the surrounding microbiome may influence disease development (Sun et al., 2023). Meanwhile, *Bacillus altitudinis* has been shown to act beneficially by protecting the host when in consortia with other bacteria such as *A. faecalis* and *B. safensis* (Dinesh et al., 2026). These bacterial behaviors depend on microbiome dysbiosis, which can alter chemical communication via quorum sensing, affecting ecological dynamics shaped by environmental stressors such as nutrient availability, farming practices, and host genotype; such factors can lead to the secretion of virulence factors or the establishment of symbiotic relationships (Ali et al., 2025; Chen et al., 2024; Zheng et al., 2025).

Whereas the current study relies on prediction tools and associations with previously reported functions and roles of these isolates, increasing sample size and incorporating broad local sampling, including asymptomatic and symptomatic fruit tissues, combined with high-throughput sequencing like Oxford Nanopore Technology, will enable more accurate bacterial genomic profiling (Basdani et al., 2026). Therefore, analyzing genomic functional annotation allows for the identification of key virulence factors, such as high resistance of *Klebsiella* to multiple antibiotics (Jiang et al., 2016), and accessory genes, thus providing a clearer understanding of their ecological dynamics (Zhang et al., 2025). These approaches will help validate the predicted functions and potential capabilities within these isolates. Furthermore, infection assays studying in vitro disease development in different model hosts (plants/human cells) can offer insights into the inter-kingdom colonization capacities of these isolates (Virgo et al., 2025). Consequently, the integrative approach of phenotypic assays with in-depth genomic analysis can yield a comprehensive view of their ecology and roles.

In conclusion, the detection of *Klebsiella* *variicola* and *Pantoea* *dispersa.* represents, to our knowledge, the first report of these bacteria isolated from *Musa* spp. fruits in Puerto Rico. Both have been associated with either beneficial or pathogenic effects, highlighting the importance of understanding their ecological impact on local *Musa* spp. systems. Therefore, further strain-level analysis and research on host-microbiome interactions are needed to assess whether Puerto Rican *K. variicola* and *P. dispersa* populations are beneficial, commensal, or potentially harmful. The identification and isolation of *Bacillus altitudinis* from symptomatic banana fruit tissues may be associated with the proposed biocontrol, given its potential antagonistic and beneficial effects suggested in prior studies. These findings open avenues for further research into application, antagonism, genomics, and virulence factors, particularly regarding potential inter-kingdom interactions. Such studies can enhance understanding of host adaptation, colonization, and ecological dynamics. Despite the study limited sampling scope, it highlights the importance of broader surveys across the island to explore bacterial diversity and distribution in *Musa* spp. fruits.

## Methods

**Isolation of bacteria from banana fruits.**

Banana fruits were obtained from a local farmer in Barranquitas, P.R., and aseptically transferred to the laboratory facilities at IAUPR-BR. Banana fruits were sampled from symptomatic (wilting/necrosis) areas using a sterile swab moistened with saline solution (0.85%) (Li et al., 2019) to isolate associated bacteria. Swab samples were streaked on Tryptic Soy Agar (TSA; Millipore, Cat. No. 22091) and incubated overnight at 30°C. Subsequently, overnight growth was purified by quadrant streaking on TSA and incubated under the same conditions.

**Phenotypic characterization**

Conventional phenotypic methods were used to gain insights and characterize the isolates. After purification, three distinct colonies were examined by describing their macro- and microscopic features, including colony morphology, Gram stain status, and catalase enzyme presence or absence. Briefly, fresh bacterial cultures were grown in Tryptic Soy Broth (TSB; Millipore, Cat. No. 22092) at approximately 30 °C ± 1 for 24 hours. Subsequently, catalase tests and Gram staining were performed. For the catalase test, a positive result was indicated by the colony's ability to release oxygen, while pink-violet bacterial cells indicated the respective Gram status. Gram stains were visualized in a compound brightfield biological microscope (Olympus CX23) at a magnification of 100X. All tests were performed according to previously established methods (Reiner, 2010; Smith & Hussey, 2019).

**DNA barcoding, phylogenetics, and functional predictions**

For DNA barcoding, samples were sent to Azenta Life Science (South Plainfield, NJ, USA) following the packaging protocol (purified isolates on Petri dishes, labeled and sealed with parafilm) for DNA isolation, PCR amplification of the 16S rDNA V1 – V9 region with Azenta Life Sciences proprietary universal primers, followed by Sanger sequencing. The resulting FASTA files were analyzed on NCBI BLAST to identify unknown bacteria. For precise identification, query coverage and identity similarity were set to >90% and ≥ 98.7%, respectively, as implemented in the EzBioCloud framework (de Souza et al., 2025). Identified isolated sequences were also deposited in NCBI and can be found under the accession numbers provided in Table 1. 

The phylogenetic tree was constructed using MEGA 12 (Kumar et al., 2018), following multiple sequence alignments with MUSCLE. Evolutionary relationships were inferred using the Maximum Likelihood method and the Tamura–Nei model (Tamura & Nei, 1993), incorporating a proportion of invariant sites (TN93+I). The model was selected as the most suitable based on the lowest Bayesian Information Criterion (BIC) values, with 1,000 bootstrap replications (Felsenstein, 1985). Sequences utilized for comparison were obtained from previously characterized phytopathogenic strains as well as non-pathogenic strains (Jena et al., 2023; Jiang et al., 2024; Kulkarni et al., 2013; Laczeski et al., 2020; Lin et al., 2015; Toh et al., 2019). The phylogenetic tree was rooted using *Micrococcus luteus* as an outgroup.

Ecological roles and functional associations of isolated bacteria were inferred based on the nearest evolutionary and taxonomic relationships between 16S rDNA amplicon sequences and reference databases, using the software Functional Annotation of Prokaryotic Taxa (FAPROTAX) (Louca et al., 2016). The prediction tool was employed as described by Sansupa et al. (2021), with minor modifications that included direct sequencing of purified isolates rather than sequencing a mixed enriched culture. The latest version of the software and database (FAPROTAX-mapper 2.0b) was employed following the recommended pipeline by the developers ( FAPROTAX v2 ). Input files included an abundance table and a FASTA file containing all three near full-length 16S rDNA amplicon sequences (~1,400 bp). A prevalence filter was applied manually to the output functional prediction file, retaining functions present in 1 or more isolates. Bubble plots were generated in R (4.5.2) in RStudio with the following packages: ggplot2 (4.0.3), dplyr (1.2.1), and tidyr (1.3.2).
